# Performance of four platforms for *KRAS* mutation detection in plasma cell-free DNA: ddPCR, Idylla, COBAS z480 and BEAMing

**DOI:** 10.1038/s41598-020-64822-7

**Published:** 2020-05-15

**Authors:** D. C. L. Vessies, M. J. E. Greuter, K. L. van Rooijen, T. C. Linders, M. Lanfermeijer, K. L. Ramkisoensing, G. A. Meijer, M. Koopman, V. M. H. Coupé, G. R. Vink, R. J. A. Fijneman, D. van den Broek

**Affiliations:** 1 Netherlands Cancer Institute, department of laboratory medicine, Amsterdam, The Netherlands; 2 Amsterdam University Medical Centers, location VUmc, department of epidemiology and biostatistics, Amsterdam, The Netherlands; 3 University Medical Center Utrecht, department of medical oncology, Utrecht University, Utrecht, The Netherlands; 4 Netherlands Cancer Institute, department of pathology, Amsterdam, The Netherlands; 5 Netherlands Comprehensive Cancer Organisation, department of research, Utrecht, The Netherlands

**Keywords:** Tumour biomarkers, Cancer genetics, Cancer genetics

## Abstract

Multiple platforms are commercially available for the detection of circulating cell-free tumour DNA (ctDNA) from liquid biopsies. Since platforms have different input and output variables, deciding what platform to use for a given clinical or research question can be daunting. This study aimed to provide insight in platform selection criteria by comparing four commercial platforms that detect *KRAS* ctDNA hotspot mutations: Bio-Rad droplet digital PCR (ddPCR), BioCartis Idylla, Roche COBAS z480 and Sysmex BEAMing. Platform sensitivities were determined using plasma samples from metastatic colorectal cancer (mCRC) patients and synthetic reference samples, thereby eliminating variability in amount of plasma analysed and ctDNA isolation methods. The prevalence of *KRAS* nucleotide alterations was set against platform-specific breadth of target. Platform comparisons revealed that ddPCR and BEAMing detect more *KRAS* mutations amongst mCRC patients than Idylla and COBAS z480. Maximum sample throughput was highest for ddPCR and COBAS z480. Total annual costs were highest for BEAMing and lowest for Idylla and ddPCR. In conclusion, when selecting a platform for detection of ctDNA hotspot mutations the desired test sensitivity, breadth of target, maximum sample throughput, and total annual costs are critical factors that should be taken into consideration. Based on the results of this study, laboratories will be able to select the optimal platform for their needs.

## Introduction

Patients with metastatic colorectal cancer (mCRC) may be treated with targeted therapies directed against epidermal growth factor receptor (*EGFR*). However, presence of a Kirsten rat sarcoma (*KRAS*) mutation in the tumour confers resistance to this type of therapy^1^. In the current standard of care the presence of *KRAS* mutations is determined in tissue biopsies obtained from the tumour. Obtaining such biopsies is invasive to the patient, may not fully represent tumour heterogeneity^2^, and is cost and time intensive. Detection of *KRAS* mutations in circulating cell-free DNA (cfDNA) from liquid biopsies offers an attractive alternative^3^. Yet, cfDNA testing has its challenges, including the small amounts of available cfDNA and low fractions of circulating tumour DNA (ctDNA)^4^. Multiple commercial ctDNA detection platforms are available, ranging from PCR based hotspot analysis to broad targeted NGS applications. These platforms show considerable differences in the amount of plasma required as input, the DNA isolation method, quantitative versus semi-quantitative results, the breadth of target and the total cost per sample analysed. These differences complicate a straightforward comparison of platforms, which results in a knowledge gap in cfDNA testing^5^. Attempts to perform such comparisons have been made^6–9^, but it cannot be excluded that the results were biased by using different amounts of plasma or cfDNA, different isolation methods^10^ and/or the use of tissue biopsy results as the gold standard. In addition these studies did not evaluate factors influencing the choice for a platform in daily practice such as the costs of analysis, the maximum annual throughput and the differences in the number of mutations targeted by a platform. Four commercially available PCR-based platforms for detection of hotspot mutations in *KRAS* (Bio-Rad droplet digital PCR (ddPCR), BioCartis Idylla, Roche COBAS z480 and Sysmex BEAMing) were compared in this study, while limiting or eliminating the impact of factors that affect a direct comparison of platforms. Furthermore the costs of analysis and the impact of the choice for a platform on detection of *KRAS* mutations in mCRC patients were investigated.

## Materials and methods

### Patient selection and blood collection

Seventeen patients with histopathologically confirmed mCRC were included between July 2017 and February 2018 through the nationwide Prospective Dutch Colorectal Cancer cohort (PLCRC)^11^. PLCRC was approved by the Medical Ethical Committee (METC) of the University Medical Center Utrecht. The review board at each participating institution approved the study, which was conducted according to the principles of the Declaration of Helsinki and the International Conference on Harmonisation Good Clinical Practice guidelines. All patients provided written informed consent to participate in the study. Patients were selected based on their *KRAS* mutation status as determined in tissue biopsies. Two patients without a *KRAS* mutation (of whom one with a *KRAS* amplification) were also included. Mutations in tissue were determined as part of routine diagnostics, using the method of choice for each including hospital. Specifically this was the Ion Torrent Hotspot panel v2plus (14×), the Therascreen KRAS extension pyro kit (1×) and unknown (2×). Clinical data for each patient at the time of liquid biopsy are summarised in Supplemental Table 1. Blood was collected at a single time point during treatment for metastatic disease in four 10 ml Cell-free DNA BCT tubes (Streck, La Vista, NE, USA) and shipped to the Netherlands Cancer Institute (NKI, Amsterdam, the Netherlands). Cell-free plasma was obtained by a two-step centrifugation protocol (10 minutes at 1700g, followed by 10 minutes at 20000 g). Cell-free plasma was stored at −80 °C.

### cfDNA isolation

CfDNA was isolated using the isolation method provided with each platform or with the QIAsymphony Circulating DNA kit (Qiagen, Düsseldorf, Germany) on the QIAsymphony (Qiagen). For the latter 4 ml of plasma was isolated and the elution volume set to 60 µl.

### Construction of synthetic reference samples

Full length genomic DNA (gDNA) (Promega, Madison, WI, USA), containing no mutations in *KRAS*, was fragmented enzymatically with dsDNA Fragmentase [#M0348] (New England Biolabs, Ipswich, MA, USA) according to manufacturer instructions. Briefly, 60 µg gDNA was incubated with dsDNA Fragmentase and incubated for 30 minutes at 37 °C, in 30 reactions of 2 µg gDNA each. The product of the 30 reactions was pooled and double-sided SPRI cleanup was performed with Agencourt AMPure XP beads [#A63881] (Beckman Coulter Life Sciences, Indianapolis, IN, USA), using 0.8x and 2.5x ratios according to manufacturer instructions. The resulting pool of cfDNA-like wildtype DNA was analysed on the Agilent 2100 BioAnalyzer system (Agilent, Santa Clara, CA, USA) using a High Sensitivity kit (#5067–4626) (Supplemental Fig. 1).

Seven synthetic DNA fragments containing mutations in the *KRAS* gene (*KRAS* p.G12A, p.G12C, p.G13D, p.A59T, p.Q61H, p.K117N or p.A146V) were ordered as gBlocks Gene Fragments with a length of 973–999 bp from IDT (Integrated DNA Technologies Inc, Skokie, IL, USA). The sequences are provided in Supplemental sequences 1. These were fragmented sonically on a Covaris ME220 Focused-ultrasonicator (Covaris inc, Woburn, MA, USA) using microTUBE AFA Fiber Pre-Slit Snap-Cap (PN 520045) vessels, with the following settings: Duration 100 s, Peak Power 75 W, Duty Factor 25% and 1000 Cycles per Burst. No BioAnalyzer results are available for the fragmented oligos, as the DNA concentration is below the limit of detection for that device. The sheared synthetic DNA fragments were pooled equimolarly and spiked into the cfDNA-like wildtype DNA to achieve mutant allele frequencies (mAF) of 0.50%, 0.04%, 0.02% and 0% (i.e. no synthetic DNA spiked, wildtype control). In total six different constructed reference samples were used in this study: 50 ng input with 0.50%, 0.02% and 0% mAF, and 10 ng input with 0.50%, 0.04% and 0% mAF. Four replicates of every constructed reference sample were measured to assess the sensitivity of each platform.

### Bio-Rad ddPCR

For Bio-Rad ddPCR the KRAS G12/G13 screening kit (#1863506, Bio-Rad) was used according to manufacturer’s instructions. All measurements were performed in duplicate, using an 18 µl sample, 2 µl ddPCR KRAS G12/G13 Screening Multiplex Assay and 22 µl ddPCR Supermix for Probes (no dUTP) (catalogue number 186–3023). Droplets were generated with QX100 Droplet Generator and measured with QX100 Droplet Reader. Data were analysed with QuantaSoft (Bio-Rad) version 1.7.4.0917. When analysing constructed reference samples containing three mutations in *KRAS* codons 12 and 13, the three mutant droplet clouds were identified and analysed independently.

For data interpretation we applied a dynamic limit of blank (LoB) that is dependent on the assay used and the concentration of the sample being analysed. The false positive rate (FPR) for the ddPCR KRAS G12/G13 Screening kit had previously been determined using 60-fold measurement of Horizon *KRAS* Wild Type Reference Standard DNA (#HD710, Horizon) at 25 and 250 copies/µl. FPR was defined as the ratio of false positive mutant molecules over wildtype molecules, and used to determine the LoB in each sample using a binomial model with 0.1% cut-off. For example, in a duplicate experiment where 6000 wildtype molecules are observed and FPR at that concentration being 10^−4^, the binomial probability for observing more than three (false positive) mutant events by chance is 0.4%, and therefore cannot be excluded as a random chance event. By contrast, if more than four mutant positive events are observed (p < 0.1%) this is considered to be a true biological signal, and the sample is interpreted as positive for that mutation.

### Idylla

Biocartis Idylla™ (Biocartis NV, Mechelen, Belgium) was used with the Idylla™ ctKRAS Mutation Test (REF A0081/6) according to manufacturer instructions unless otherwise indicated. Where previously isolated DNA was used with Idylla, it was diluted in nuclease free H_2_O (NF-H_2_O) to 1 ml and loaded onto the cartridge. This procedure was previously determined to not impact the performance of the system negatively (data not shown). Results were obtained and analysed in the IdyllaExplore environment, allowing for the identification of multiple mutations per sample.

### COBAS z480

Roche COBAS z480 (Roche Molecular System Inc, Pleasanton, CA, USA) was used with the *KRAS* Mutation Test v2 LSR kit (material number 07989270001) according to manufacturer instructions unless otherwise indicated. Where previously isolated DNA was used with COBAS z480, it was diluted in NF-H_2_O to 70 µl prior to PCR setup. Data was analysed according to instructions by uploading the.ixo files to the online LSR Data Analysis tool (https://lifescience.roche.com/en_nl/brands/oncology-research-kits.html).

### BEAMing

Sysmex Inostics BEAMing Digital PCR (Sysmex Inostics GmbH, Hamburg, Germany) was used with the OncoBEAM™ RAS CRC kit RUO (ZR150001) and the CyFlow Cube 6i and Robby instruments according to manufacturer instructions unless otherwise indicated. Where previously isolated DNA was used with BEAMing, it was diluted in NF-H_2_O to 123 µl prior to pre-amplification. Data was analysed for the *KRAS* variants only (ignoring *NRAS* variants), using the BEAMing software according to instructions.

Technical performance data for all four platforms are provided in Supplemental Table 2.

### Breadth of target

The point mutations in *KRAS* that are targeted by each platform were evaluated from the respective product specifications. These were compared to publicly available tissue biopsy mutation profiles for 1099 mCRC patients^12^, that were accessed through the cBioPortal for Cancer Genomics^13,14^ on December 14^th^, 2018.

### Total annual costs

We determined the total annual cost according to the Activity Based Costing (ABC) model^15^, including all reagents costs, hands-on time costs, maintenance costs and depreciation costs for all equipment used. The material costs include costs for cfDNA isolation, kit costs, control samples and additional materials. Hands-on time per sample was determined for two scenario’s: High throughput (maximum number of samples per week based on maximal occupancy of the machine) and low throughput (5 samples per week). Intermediate throughput was modelled by linear interpolation of those results. Equipment depreciation was calculated by applying an annuity factor based on equipment depreciation in 10 years with an interest rate of 4.2%. Maintenance was incorporated by applying a fixed annual cost for maintenance contracts for each platform. Costs were included as raw list price costs, including all relevant taxes and were analysed as a function of annual sample throughput for each platform. To determine what factors have a large or small effect on the total cost per year we performed cost sensitivity analyses for the following parameters: 1) Equipment depreciation in 5 years rather than 10 years and 2) Manual cfDNA isolation for ddPCR, with the QIAamp Circulating Nucleic Acid Kit (Qiagen) rather than the QIAsymphony.

## Results

**Figure 1:**
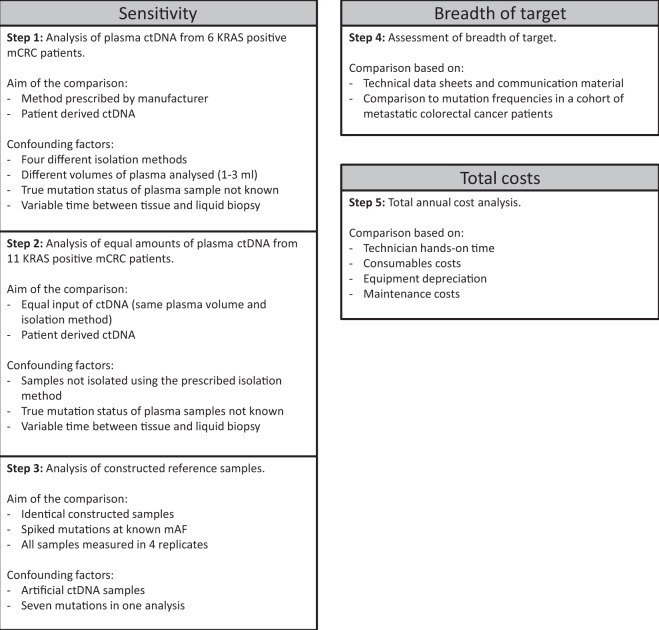
Graphical representation of the five step approach for comparison of four commercially available ctDNA platforms.

The experimental set-up to determine the sensitivity of each platform is shown in Fig. 1, steps one to three. cfDNA from six mCRC patients was analysed following the manufacturer’s instructions as indicated in the first step of Fig. 1. Tissue mutation analysis was performed as part of routine clinical care and in five of the six patients a *KRAS* mutation was reported. The time between the tissue analysis and the collection of the plasma ranged from 0 to 39 months. The amount of isolated cfDNA ranged from 4.3 to 53.1 ng/ml plasma. In two out of five *KRAS* positive patients all platforms detected the *KRAS* mutation. For two *KRAS* positive patients a *KRAS* mutation was detected by three of the four platforms and in one *KRAS* positive patient no *KRAS* mutation was detected in plasma by any of the platforms. For the sixth patient, for whom tissue analysis did not identify a *KRAS* mutation, two platforms did report a *KRAS* mutation. Results are shown in Table 1.

**Table 1:** Mutations detected by four commercially available ctDNA detection platforms in cfDNA from 6 patients with mCRC and corresponding tissue results.

Tissue *KRAS* result^a^	Months between tissue biopsy and blood collection	Concentration cfDNA in plasma (ng/ml)^b^	Reported mutant copies (mAF)^c^	ddPCR^d^	Idylla^d^	COBAS z480^e^	BEAMing^f^
*KRAS* wildtype	39	4.3	75 (0.24%)	nd^g^	*KRAS* p.G12R	nd	*KRAS* p.G12X^h^
*KRAS* p.G12S	10	5.5	nd	nd	nd	nd	nd
*KRAS* detected	Unknown	53.1	49 (0.05%)	*KRAS* p.G12X/G13X^i^	*KRAS* p.G12S	nd	*KRAS* p.G12X
*KRAS* p.G12D	1	14.1	5323 (12.51%)	*KRAS* p.G12X/G13X	*KRAS* p.G12D	*KRAS* p.G12X	*KRAS* p.G12X
*KRAS* p.G12D	0	5.6	172 (0.67%)	*KRAS* p.G12X/G13X	nd	*KRAS* p.G12X	*KRAS* p.G12X
*KRAS* p.G12S	4	17.0	279 (0.62%)	*KRAS* p.G12X/G13X	*KRAS* p.G12S	*KRAS* p.G12X	*KRAS* p.G12X

^a^Tissue KRAS result is based on the standard of care test at the hospital of inclusion.

^b^cfDNA concentration in plasma is based on Qubit measurement of cfDNA isolated by QIAsymphony using the Circulating DNA kit, and is reported in ng cfDNA per ml plasma isolated.

^c^Reported mutant copies and mutant allele frequency (mAF) are based on BEAMing results.

^d^Results are based on 1 ml of plasma.

^e^Results are based on 2 ml of plasma.

^f^Results are based on 3 ml of plasma.

^g^nd = not detected

^h^KRAS p.G12X = any variant at amino acid position G12 of the KRAS gene. The result is not further specified by BEAMing or COBAS z480 platforms.

^i^KRAS p.G12X/G13X = any variant at amino acid positions G12 or G13 of the KRAS gene. The result is not further specified by ddPCR platform.

### Analysis of patient derived cfDNA at equal inputs per platform

A number of confounding factors could have influenced the results from the comparison per manufacturer’s instructions, including different volumes of plasma and different cfDNA isolation methods used (Fig. 1). Analysis of cfDNA from 11 mCRC patients with tissue-confirmed *KRAS* mutations using a single isolation method and distributing the DNA equally over the platforms allowed us to eliminate these differences, indicated in Fig. 1 step two. Time between tissue biopsy and liquid biopsy ranged from 0 to 22 months. The amount of isolated cfDNA ranged from 4.7 to 185.6 ng/ml plasma. In six out of 11 patients (54%) the results from all four platforms were concordant. *KRAS* p.A146T in one patient was detected by all platforms with the exception of ddPCR which did not target this mutation. Idylla reported a *KRAS* mutation in two patients, concordant with the *KRAS* mutation that had been detected in tissue, which was not detected by the other platforms. In two patients the mutations (*KRAS* p.G12_G12insAG and a *KRAS* amplification) were not targeted by any platform, but BEAMing did report the presence of a *KRAS* p.G12X mutation for the patient with a *KRAS* p.G12_G12insAG mutation. The *KRAS* amplification was not detected by any of the platforms as expected. When 10 ng or more input of cfDNA (n = 8) was used, the detected *KRAS* mutations were concordant across all platforms when considering only mutations that can be detected by all platforms. For five samples we could determine the mAF of the *KRAS* mutation by ddPCR and/or BEAMing. These ranged from 0.12%-15.4% (9–4656 copies/ml) (Table 2). All variants detected by all platforms were present with 39 mutant copies per platform or more.

**Table 2:** Mutations detected by four commercially available ctDNA detection platforms in cfDNA from 11 patients with mCRC isolated with the QIAsymphony and distributed equally over all platforms.

Tissue *KRAS* result^a^	Months between tissue biopsy and blood collection	total cfDNA analysed (ng)^b^	Reported mutant copies (mAF)^c^	ddPCR	Idylla	COBAS z480	BEAMing
*KRAS* p.G12_G12insAG	Unknown	23.7	9 (0.12%)	nt^d^	nt	nt	*KRAS* p.G12X
*KRAS* p.G12V	Unknown	23.0	1167 (15.4%)	*KRAS* p.G12X/G13X	*KRAS* p.G12V	*KRAS* p.G12X	*KRAS* p.G12X
*KRAS* p.A146T	9	17.3	106 (1.7%)	nt	*KRAS* p.A146T	*KRAS* p.146×	*KRAS* p.A146T
*KRAS* p.G12D	0	94.4	39 (0.13%)	*KRAS* p.G12X/G13X	*KRAS* p.G12D	*KRAS* p.G12X	*KRAS* p.G12X
*KRAS* p.G13D	Unknown	8.1	nd	nd^e^	*KRAS* p.G13D	nd	nd
*KRAS* p.G12V	Unknown	167.0	4656 (8.5%)	*KRAS* p.G12X/G13X	*KRAS* p.G12V	*KRAS* p.G12X	*KRAS* p.G12X
*KRAS* p.G12C	5	10.1	nd	nd	nd	nd	nd
*KRAS* p.G12D	9	13.7	nd	nd	nd	nd	nd
*KRAS* p.G12D	20	8.2	nd	nd	*KRAS* p.G12D	nd	nd
*KRAS* p.G13D	22	10.2	nd	nd	nd	nd	nd
*KRAS* amplification	Unknown	4.2	nd	nt	nt	nt	nt
Overall sensitivity (%)				38%^f^	67%	44%	50%

^a^The KRAS mutation status was determined in tissue using the method of choice of the hospital of inclusion.

^b^Total cfDNA input per method is based on Qubit measurement of cfDNA isolated by QIAsymphony using the Circulating DNA kit.

^c^Reported mutant copies and mutant allele frequency (mAF) are based on BEAMing results.

^d^nt = not targeted.

^e^nd = not detected.

^f^detected mutations divided by the total number of mutations detectable by that platform.

### Sensitivity of *KRAS* detection based on synthetic reference samples

Analysing four replicates of synthetic reference samples harbouring multiple *KRAS* mutations allowed us to eliminate the effect of not knowing the true mutation status of the samples, and limit the effects of sampling errors due to replicate measurements, as outlined in step three of Fig. 1. Overall, more mutations were detected at higher total input and higher mAF, validating the successful construction of the synthetic reference samples. At 10 ng DNA input valid results were obtained for 0% and 38% of COBAS z480 and BEAMing measurements, respectively. At 50 ng this increased to 83% and 93% of measurements, respectively. ddPCR and Idylla did not report any invalid results. The sensitivity depended on the total amount of input for each platform. At a mAF of 0.5%, 62 mutations were detected with 50 ng input, compared to 29 mutations for 10 ng input. At 50 ng input the COBAS z480 reported a *KRAS* p.A59X variant in all valid replicates of the wildtype control samples. The percentage of all mutations detected ranged from 39% (BEAMing) to 13% (Idylla) (Table 3).

**Table 3:** Mutations detected in constructed reference samples by four commercially available ctDNA detection platforms.

Input DNA (ng)	Mutant allele frequency (%)	Mutant copies per analysis^a^	ddPCR		Idylla		COBAS z480^b^		BEAMing^c^	
			All mutations (%)	*KRAS* p.G12/G13 (%)	All mutations (%)	*KRAS* p.G12/G13 (%)	All mutations (%)	*KRAS* p.G12/G13 (%)	All mutations (%)	*KRAS* p.G12/G13 (%)
10	0.00^d^	0	nd^e^	nd	nd	nd	nd	nd	nd	nd
10	0.04	1	nd	nd	nd	nd	nd	nd	4^f^	12
10	0.50	15	43	100	11	16	nd	nd	54	62
50	0.00^d^	0	nd	nd	nd	nd	12^g^	nd	nd	nd
50	0.02	3	25	58	4	8	17	nd	8	25
50	0.50	75	43	100	36	50	62	38	92	75
Overall sensitivity (%)			28	65	13	19	20	10	39	44
Sensitivity 50 ng (%)			34	79	20	29	40	19	50	50
Sensitivity 10 ng (%)			22	50	6	8	0	0	27	37

^a^Average number of mutant copies per analysis was calculated as Input DNA (ng) * 300 (Genome Equivalents/ng) * Mutant allele frequency (Mutant copies/Genome Equivalent).

^b^For COBAS z480, invalid results were obtained at 10 ng DNA input for all replicates, at 50 ng of DNA input invalid results were obtained in 25% of the replicates at 0 and 75 mutant copies. Invalid results were counted as not detected.

^c^For BEAMing, invalid results were obtained at 10 ng DNA input in 88%, 54% and 46% of the replicates at 0, 1 and 15 mutant copies respectively. At 50 ng input 7% of all replicates were reported as invalid. Invalid results were counted as not detected.

^d^Wildtype control samples without synthetic mutant fragment spike-in.

^e^nd* =* not detected.

^f^Detected mutations divided by the total number of mutations present over four replicates. Not all platforms target all mutations, and will have lower reported sensitivity as a result.

^g^A false positive KRAS A59X was reported in all wildtype replicates. These false positives was based on a software error (personal communication with Roche Diagnostics).

For comparison of platform sensitivities we evaluated a subset of three mutations (*KRAS* p.G12A, p.G12C and p.G13D) that were targeted by all four platforms. Sensitivity over all mAFs ranged from 10% (COBAS z480) to 65% (ddPCR). Considering only samples with 0.5% mAF (15 and 75 mutant copies/reaction) sensitivities ranged between 19% (COBAS z480) and 100% (ddPCR) (Table 3). Raw reported mutation detection values are provided in Supplemental data set 1.

### Impact of breadth of target detection

To determine the impact of having a broader panel when analysing cfDNA, the number of mutations targeted per platform were compared to publicly available tissue biopsy mutation profiles of 1099 mCRC patients^12^. Of 1099 patients, 46% (505/1099) had a mutation in *KRAS*. ddPCR targets 82% of those (413/505), Idylla and COBAS z480 both 96% (485/505) and BEAMing 94% (477/505). To estimate the effect of platform sensitivity superimposed on platform breadth on the detection of *KRAS* mutations in a general mCRC population, the sensitivities determined on synthetic reference samples at 50 ng input with 0.5% or 0.02% mAF were included. Based on these assumptions ddPCR and BEAMing were likely to detect *KRAS* mutations at a mAF of 0.5% in respectively 38% and 32% of mCRC patients, compared to 22% and 17% for Idylla and COBAS z480. At 0.02% mAF, ddPCR showed to detect 22% of patients, Idylla 8%, COBAS z480 0%, and BEAMing 11% (Table 4).

**Table 4:** Estimation of the impact of the breadth of target and sensitivity of platforms at two different mutant allele fraction levels in the detection of KRAS mutations in a mCRC population.

	Prevalence of *KRAS* mutations in mCRC	Breadth of *KRAS* targets	Sensitivity at 50 ng DNA mAF 0.50%^a^	Sensitivity at 50 ng DNA mAF 0.02%^a^	Estimated % of *KRAS* positive patients detected (50 ng, mAF 0.50%)	Estimated % of *KRAS* positive patients detected (50 ng, mAF 0.02%)
ddPCR	46%	82%	100%	58%	38%	22%
Idylla	46%	96%	50%	8%	22%	4%
COBAS z480	46%	96%	38%^b^	0%	17%	0%
BEAMing	46%	94%	75%^c^	25%^d^	32%	16%

^a^Sensitivity was based on the detection of KRAS p.G12 and p.G13 mutations in synthetic reference samples at the indicated input and mAF.

^b^Invalid result were obtained for 25% of the results.

^c^Invalid result were obtained for 25% of the results.

^d^Invalid result were obtained for 12% of the results.

### Total cost analysis

**Figure 2:**
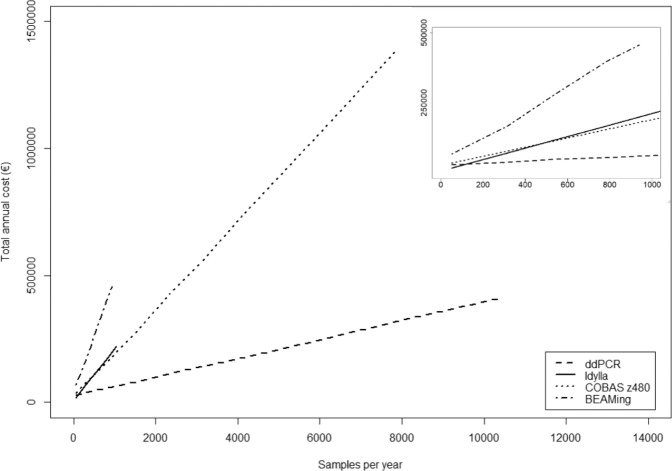
Total annual costs as a function of the number of samples analysed per year. The width on the x-axis is determined by the maximum number of samples that can be analysed per year based on optimal platform occupancy.

The total annual cost of the platforms correlated linearly to the number of samples analysed per year (R^2^ for linearity between 0.9973 and 1.000) (Fig. 2 and Supplemental data set 2). Total annual costs were highest for BEAMing, while ddPCR was found to be the least expensive platform to use when more than 110 samples were analysed per year. At lower throughput Idylla was found to be slightly less expensive due to lower fixed annual costs.

For all platforms, the material costs per sample were the largest contributor to the total annual costs. The higher the throughput the greater the relative contribution of the material costs became for all platforms, up to 80% for BEAMing (940 samples per year) and 95% for COBAS z480 (7800 samples per year) (Supplemental data set 2).

Given the rapidly developing field of ctDNA detection, the impact of an instrument depreciation time of 5 instead of 10 years was evaluated. This increased the fixed annual costs with 31% (COBAS z480) to 73% (Idylla). A limited effect of using manual cfDNA isolation with the QIAamp Circulating DNA Kit versus automated cfDNA isolation with QIAsymphony was observed (Supplemental data set 2).

## Conclusion/discussion

We show that performing a systematic comparison is complicated by multiple factors, all of which can impact the sensitivity. By understanding, eliminating or limiting these factors an unbiased comparison of the four platforms was performed, showing that ddPCR and BEAMing have a higher sensitivity for *KRAS* hotspot mutations than Idylla and COBAS z480. In addition it was shown that Idylla has the lowest annual cost at low sample throughput, while ddPCR is least expensive at higher sample throughput. BEAMing is the most expensive platform overall.

To compare the sensitivity of each platform in this study a number of factors were considered: The volume of plasma used, the total DNA input, and the isolation method. By performing the sensitivity comparison in three steps we could eliminate the impact of each of these factors. In the first step 6 patient samples were analysed following the protocols of the respective platforms (Table 1). Several factors could have influenced these results, hampering a direct link to the performance of the platforms (Fig. 1). To eliminate possible effects from different plasma volumes and isolation methods, patient plasma was isolated using a single method and the isolated cfDNA was distributed equally over the platforms. The results were fully concordant for samples with at least 39 mutant copies per reaction, which was in line with results obtained with synthetic reference samples. Compared to the other platforms Idylla detected two additional mutations in samples with less than 8 ng cfDNA input (Table 2). Since Idylla does not report mAFs we cannot exclude that this is the result of sampling distribution errors. All patient samples in this study were obtained during treatment, and time between tissue and liquid biopsy differed greatly between patients. This complicates the interpretation of the results, as the true mutation status of these plasma samples is unknown at the time of the liquid biopsy.

In order to limit the effects of sampling errors and eliminate the unknown true mutation status of patient plasma cfDNA, synthetic reference samples were constructed and measured in four replicates. ddPCR and BEAMing performed better than Idylla and COBAS z480 (Table 3), both overall and among a limited number of mutations that were targeted by all platforms. This is in line with previous reports^6–9^.

Sensitivity is not the only factor that defines the performance of a platform. The detection of *KRAS* mutations in a real life mCRC population will depend on the sensitivity of the platform, the prevalence of specific *KRAS* mutations in the population, and the number of mutations analysed by the platform. Since few publications report the mAF of *KRAS* mutations detected in the cfDNA of mCRC cohorts^16–18^, some assumptions were required to extrapolate the data from the synthetic reference samples to a general mCRC cohort. Here we assumed 0.50% mAF and 50 ng cfDNA input, leading to a predicted detection rate of *KRAS* mutations amongst a total mCRC patient population of 17–22% for the non-digital platforms, versus 32–38% for the digital platforms (Table 4). The main factor driving this difference was the sensitivity of BEAMing and ddPCR, while the breadth of target and mutation prevalence in the target population had a more limited impact. Although no cohort of mCRC patients will have exactly 0.50% mAF and 50 ng cfDNA input, this example still provides insight in the interplay between sensitivity, breadth of target and prevalence of the mutations in the intended population, thereby further aiding future users in comparing these platforms.

For application of a platform in daily practice the total annual costs are a highly relevant factor. The costs per platform differed greatly. At low sample throughput Idylla was least expensive, while ddPCR was less expensive for higher throughput. BEAMing was the most expensive across the whole range of throughput investigated. Out of the factors investigated, the material cost per sample was the largest contributor to the total annual cost.

Overall the effectiveness of a platform to detect mutations in a patient population depends on its performance characteristics. The performance of a platform is affected by sensitivity - which depends on the amount of plasma analysed, isolation method, and PCR technique -, the character of the result (quantitative or qualitative), the number of mutations targeted, the population under investigation, and the cost of analysis. The decision which platform to use in a specific clinical or research setting will often be based on the expected population and number of samples, and the performance of the platforms in the intended situation. A direct comparison of the platforms is hampered by the lack of a gold standard and any harmonisation between the platforms.

A number of studies have compared cell-free DNA mutation detection platforms^6–9^. For example, *Garcia et al*.^6^ reported the highest sensitivity for BEAMing in a comparison of BEAMing, ddPCR and an NGS approach. In this comparison the amount of total cfDNA input for BEAMing (123 µl) was substantially higher than for ddPCR (8 µl) and NGS (10 µl). Since the amount of plasma or cfDNA analysed will affect sensitivity of the analysis this might have introduced a bias. *Vivancos et al*.^7^ reported increased detection of *KRAS* mutations in a comparison of BEAMing and BioCartis Idylla. In this study BEAMing was used to select *KRAS* positive samples to be tested on the Idylla platform. By re-testing samples, different volumes of plasma were analysed (3 ml vs 1 ml). Furthermore, samples that were negative by BEAMing were not tested using Idylla, introducing a bias by design of the study. *Thress et al*.^8^ found higher sensitivity for two digital platforms (BEAMing and ddPCR) than for two non-digital platforms (COBAS and Therascreen). In this case equal volumes of plasma were used for all platforms, but having used mutations detected in tissue as the sole reference value to calculate sensitivity and specificity might still introduce discrepancies and/or biases. *Wang et al*.^9^ compared ddPCR and ARMS, finding higher sensitivity for the digital approach (ddPCR). In this study the amount of cfDNA used for each platform was not specified, complicating the interpretation of their results. Apart from the four platforms compared in this study, other methods for the detection of mutations in cfDNA are available. Further research using patient samples, equal input and reference samples as well as total cost analyses will be required to learn how other platforms compare to the platforms included in this study.

In conclusion, our results show that multiple factors affect the performance of a specific platform in daily practice. For the detection of *KRAS* mutations in a cohort of mCRC patients, the sensitivity of a platform was the most important differentiating factor compared to the number of mutations targeted and their prevalence in the target population. Idylla was the least expensive platform at low throughput, while ddPCR was less expensive at higher annual sample throughput. BEAMing was the most expensive across the whole range investigated. Selecting an optimal platform depends on the patient or study population, the yearly sample throughput, the required sensitivity in relation to the clinical or scientific question at hand and available funds.

## Supplementary information

Supplemental tables, figures and sequences.

Supplemental dataset 1.

Supplemental dataset 2.

## Data Availability

All data generated during this study are included in this published article (and its Supplemental Information files), and/or are available from the corresponding author on reasonable request.
