# Supplementary material for: Performance of four platforms for KRAS mutation detection in plasma cell-free DNA: ddPCR, Idylla, COBAS z480 and BEAMing
Source: Sci Rep. 2020 May 15;10:8122. doi: 10.1038/s41598-020-64822-7 (PMC7229219; doi:10.1038/s41598-020-64822-7)
Supplement: Supplementary file 1 — Supplemental tables, figures and sequences. [file 41598_2020_64822_MOESM1_ESM.docx]

| ***Supplemental table 1.*** *Clinical data for patients presented in tables 1 and 2.* | | | | | | | |
| --- | --- | --- | --- | --- | --- | --- | --- |
| **Patient ID (table/**  **line)** | **Palliative systemic treatment agents** | **Palliative systemic treatment line** | **Previous systemic treatments** | **Time on treatment (months)** | **Disease status** | **First response evaluation after liquid biopsy** | **Metastatic site(s)** |
| 1/1 | encorafenib/  binimetinib/ cetuximab | 3rd line | CAPOX (6x), CAP (4x), irinotecan (3x) | 3 | Partial response | RECIST PR | Liver, lymph nodes |
| 1/2 | CAP-B (maintainance) | 1st line | Started CAPOX-B (6x), CAP-B maintainance | 9 | Signs of progression | RECIST PD | Peritoneum |
| 1/3 | FOLFIRI-B | 1st line | Adjuvant CAPOX (8x) | 0.5 | Recently started systemic therapy | RECIST SD, tumor load increasing | Lymph nodes, peritoneum |
| 1/4 | FOLFOX-B | 1st line | None | 0.5 | Recently started systemic therapy | RECIST PD | Liver, lymph nodes |
| 1/5 | start FOLFOXIRI-B | 1st line | None | 0 | At start palliative systemic treatment | RECIST SD, tumor load not altered | Liver, peritoneum, lung |
| 1/6 | FOLFOXIRI-B | 1st line | None | 2 | Recently started systemic therapy | RECIST SD, tumor load decreasing | Liver |
| 2/1 | Trifluridine-tipiracil | 3rd line | CAPOX/FOLFOX (10x), CAP (6x), irinotecan (2x) | 0.5 | Recently started new systemic therapy | RECIST PD | Liver, lymph nodes, lung, peritoneum, subcutis |
| 2/2 | CAP-B (maintainance) | 1st line | CAPOX-B (2x), CAP-B reintroduction after treatment holiday | 0 | At start reintroduction CAP-B | RECIST SD, tumor load decreasing | Liver, bones, local recurrence primary tumor |
| 2/3 | CAP-B | 2nd line | CAPOX-B (6x), FOLFIRI (metastasectomy thereafter) | 1 | Recently started new systemic therapy | RECIST PD | Liver, lymph nodes |
| 2/4 | Not started, started CAP-B 2 months later | n.a. | None | 0 | Treatment naive, started CAP-B 2 months later | n.a. | Liver |
| 2/5 | CAP-B | 1st line | None | 3 | Responsive to firstline treatment | RECIST SD, tumor load decreasing | Liver, lung, peritoneum |
| 2/6 | FOLFOX-B | 1st line | None | 0 | At start palliative systemic treatment | RECIST SD, tumor load not altered | Liver, lung, lymph nodes, primary tumor |
| 2/7 | CAP-B (maintainance) | 1st line | CAPOX-B (4x) | 5 | Stable disease | RECIST SD, tumor load not altered | Liver, lung, primary tumor |
| 2/8 | FOLFIRI-B | 1st line | None | 1 | Recently started systemic therapy | RECIST PR (metastasectomy was performed) | Liver |
| 2/9 | CAP-B (maintainance) | 1st line | Started CAPOX-B (6x), CAP-B maintainance | 18 | Responsive to firstline treatment | RECIST SD, tumor load increasing | Lung, primary tumor |
| 2/10 | FOLFIRI-B | 1st line | None | 2 | Stable disease | RECIST SD, tumor load not altered | Liver, peritoneum |
| 2/11 | FOLFOXIRI-B | 1st line | CAPOX-B (4x), CAPOX (4x) and tumor debulking in ORCHESTRA | 0 | Recently started new systemic therapy | RECIST PR | Lung, bones |

| ***Supplemental table 2.*** *Technical specifications of the compared platforms, as reported by manufacturers.* | | | | |
| --- | --- | --- | --- | --- |
|  | **ddPCR** | **Idylla** | **COBAS z480** | **BEAMing** |
| **Basic principle** | droplet digital PCR | quantitative PCR | quantitative PCR | droplet multiplex PCR |
| **Number of amino acid changes in KRAS detectable by platform** | 7 | 18 | 21 | 14 |
| **Plasma input volume** | None specified | 1 ml plasma | 2 ml plasma | 3 ml plasma |
| **cfDNA isolation method** | None specified | In cartridge | Column based, supplied with kit | Column based, supplied with kit |
| **Quantitation** | Quantitative  (copies/µl) | Semi-quantitative  (Cq values) | Semi-quantitative (SQI values) | Quantitative (mutant fraction) |
| **Reported lower limit of detection** | 0.1% allele frequency | 100 copies/ml plasma | 200 copies/ml plasma | 0.02% allele frequency |
| **Hands-on time** | 1-3 hours | 15 minutes | 1-3 hours | 8-9 hours |
| **Time to result** | None specified | 2-3 hours | 1-2 days | 3 days |

**
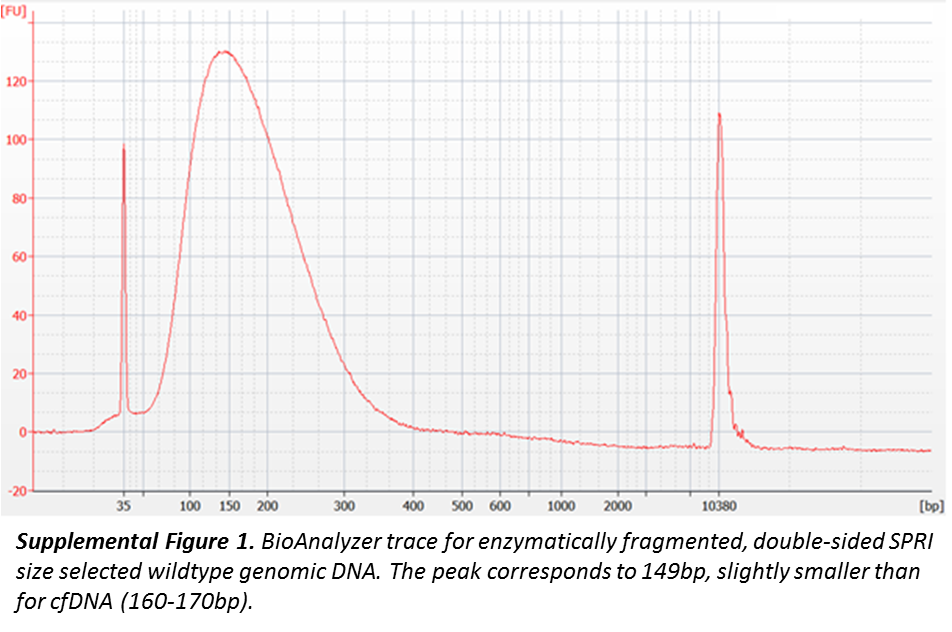
**

***Supplemental sequence 1.*** *Sequences of DNA fragments with mutation as spiked into reference samples.*

*KRAS* p.G12A. Fragment length: 999 base pairs

GAGCACTGTGAAGTCTCTACATGAGTGAAGTCATGATATGATCCTTTGAGAGCCTTTAGCCGCCGCAGAACAGCAGTCTGGCTATTTAGATAGAACAACTTGATTTTAAGATAAAAGAACTGTCTATGTAGCATTTATGCATTTTTCTTAAGCGTCGATGGAGGAGTTTGTAAATGAAGTACAGTTCATTACGATACACGTCTGCAGTCAACTGGAATTTTCATGATTGAATTTTGTAAGGTATTTTGAAATAATTTTTCATATAAAGGTGAGTTTGTATTAAAAGGTACTGGTGGAGTATTTGATAGTGTATTAACCTTATGTGTGACATGTTCTAATATAGTCACATTTTCATTATTTTTATTATAAGGCCTGCTGAAAATGACTGAATATAAACTTGTGGTAGTTGGAGCTGCTGGCGTAGGCAAGAGTGCCTTGACGATACAGCTAATTCAGAATCATTTTGTGGACGAATATGATCCAACAATAGAGGTAAATCTTGTTTTAATATGCATATTACTGGTGCAGGACCATTCTTTGATACAGATAAAGGTTTCTCTGACCATTTTCATGAGTACTTATTACAAGATAATTATGCTGAAAGTTAAGTTATCTGAAATGTACCTTGGGTTTCAAGTTATATGTAACCATTAATATGGGAACTTTACTTTCCTTGGGAGTATGTCAGGGTCCATGATGTTCACTCTCTGTGCATTTTGATTGGAAGTGTATTTCAGAGTTTCGTGAGAGGGTAGAAATTTGTATCCTATCTGGACCTAAAAGACAATCTTTTTATTGTAACTTTTATTTTTATGGGTTTCTTGGTATTGTGACATCATATGTAAAGGTTAGATTTAATTGTACTAGTGAAATATAATTGTTTGATGGTTGATTTTTTTAAACTTCATCAGCAGTATTTTCCTATCTTCTTCTCAACATTAGAGAACCTACAACTACCGGATAAATTTTACAAAATGAATTATTTGCCTAAGGTGTGGT

*KRAS* p.G12C. Fragment length: 999 base pairs

GAGCACTGTGAAGTCTCTACATGAGTGAAGTCATGATATGATCCTTTGAGAGCCTTTAGCCGCCGCAGAACAGCAGTCTGGCTATTTAGATAGAACAACTTGATTTTAAGATAAAAGAACTGTCTATGTAGCATTTATGCATTTTTCTTAAGCGTCGATGGAGGAGTTTGTAAATGAAGTACAGTTCATTACGATACACGTCTGCAGTCAACTGGAATTTTCATGATTGAATTTTGTAAGGTATTTTGAAATAATTTTTCATATAAAGGTGAGTTTGTATTAAAAGGTACTGGTGGAGTATTTGATAGTGTATTAACCTTATGTGTGACATGTTCTAATATAGTCACATTTTCATTATTTTTATTATAAGGCCTGCTGAAAATGACTGAATATAAACTTGTGGTAGTTGGAGCTTGTGGCGTAGGCAAGAGTGCCTTGACGATACAGCTAATTCAGAATCATTTTGTGGACGAATATGATCCAACAATAGAGGTAAATCTTGTTTTAATATGCATATTACTGGTGCAGGACCATTCTTTGATACAGATAAAGGTTTCTCTGACCATTTTCATGAGTACTTATTACAAGATAATTATGCTGAAAGTTAAGTTATCTGAAATGTACCTTGGGTTTCAAGTTATATGTAACCATTAATATGGGAACTTTACTTTCCTTGGGAGTATGTCAGGGTCCATGATGTTCACTCTCTGTGCATTTTGATTGGAAGTGTATTTCAGAGTTTCGTGAGAGGGTAGAAATTTGTATCCTATCTGGACCTAAAAGACAATCTTTTTATTGTAACTTTTATTTTTATGGGTTTCTTGGTATTGTGACATCATATGTAAAGGTTAGATTTAATTGTACTAGTGAAATATAATTGTTTGATGGTTGATTTTTTTAAACTTCATCAGCAGTATTTTCCTATCTTCTTCTCAACATTAGAGAACCTACAACTACCGGATAAATTTTACAAAATGAATTATTTGCCTAAGGTGTGGT

*KRAS* p.G13D. Fragment length: 999 base pairs

GAGCACTGTGAAGTCTCTACATGAGTGAAGTCATGATATGATCCTTTGAGAGCCTTTAGCCGCCGCAGAACAGCAGTCTGGCTATTTAGATAGAACAACTTGATTTTAAGATAAAAGAACTGTCTATGTAGCATTTATGCATTTTTCTTAAGCGTCGATGGAGGAGTTTGTAAATGAAGTACAGTTCATTACGATACACGTCTGCAGTCAACTGGAATTTTCATGATTGAATTTTGTAAGGTATTTTGAAATAATTTTTCATATAAAGGTGAGTTTGTATTAAAAGGTACTGGTGGAGTATTTGATAGTGTATTAACCTTATGTGTGACATGTTCTAATATAGTCACATTTTCATTATTTTTATTATAAGGCCTGCTGAAAATGACTGAATATAAACTTGTGGTAGTTGGAGCTGGTGACGTAGGCAAGAGTGCCTTGACGATACAGCTAATTCAGAATCATTTTGTGGACGAATATGATCCAACAATAGAGGTAAATCTTGTTTTAATATGCATATTACTGGTGCAGGACCATTCTTTGATACAGATAAAGGTTTCTCTGACCATTTTCATGAGTACTTATTACAAGATAATTATGCTGAAAGTTAAGTTATCTGAAATGTACCTTGGGTTTCAAGTTATATGTAACCATTAATATGGGAACTTTACTTTCCTTGGGAGTATGTCAGGGTCCATGATGTTCACTCTCTGTGCATTTTGATTGGAAGTGTATTTCAGAGTTTCGTGAGAGGGTAGAAATTTGTATCCTATCTGGACCTAAAAGACAATCTTTTTATTGTAACTTTTATTTTTATGGGTTTCTTGGTATTGTGACATCATATGTAAAGGTTAGATTTAATTGTACTAGTGAAATATAATTGTTTGATGGTTGATTTTTTTAAACTTCATCAGCAGTATTTTCCTATCTTCTTCTCAACATTAGAGAACCTACAACTACCGGATAAATTTTACAAAATGAATTATTTGCCTAAGGTGTGGT

*KRAS* p.A59T. Fragment length: 999 base pairs

ATAAATAGTGCTGCTGCGAACATTGGTGTACATGTATCTGTTTGAGTCCCTGTTTTTAGTTATTTTGGTTATATACCTAGGAATGGAATTGCTGATCATATGGTAATTCTGTGTTTAACTTTTTGAGGAACTACCACTGTTTTCCACAATGGCATCACCATTTTACATTCCCACCAGCAATGCACAAAGATTTCAGTGTCTGTATCCTTGCTAACACTTATTTTCCATTTTTTGAGTTTTTTTGTTTTGTTTTTTTAATAATAGCCAATCCTAATGGGTATGTGGTAGCATCTCATGGTTTTGATTTTATTTTCCTGACTATTGATGATGTTGAGCATCTTTTCAGGTGCTTAGTGGCCATTTGTCCGTCATCTTTGGAGCAGGAACAATGTCTTTTCAAGTCCTTTGCCCATTTTTAAATTGAATTTTTTGTTGTTGAGTTGTATATAACACCTTTTTTGAAGTAAAAGGTGCACTGTAATAATCCAGACTGTGTTTCTCCCTTCTCAGGATTCCTACAGGAAGCAAGTAGTAATTGATGGAGAAACCTGTCTCTTGGATATTCTCGACACAACAGGTCAAGAGGAGTACAGTGCAATGAGGGACCAGTACATGAGGACTGGGGAGGGCTTTCTTTGTGTATTTGCCATAAATAATACTAAATCATTTGAAGATATTCACCATTATAGGTGGGTTTAAATTGAATATAATAAGCTGACATTAAGGAGTAATTATAGTTTTTATTTTTTGAGTCTTTGCTAATGCCATGCATATAATATTTAATAAAAATTTTTAAATAATGTTTATGAGGTAGGTAATATCCCTGTTTTATAAATGAAGTTCTTGGGGGATTAGAGCAGTGGAGTAACTTGCTCCAGACTGCATCGGTAGTGGTGGTGCTGGGATTGAAACCTAGGCCTGTTTGACTCCACAGCCTTCTGTACTCTTGACTATTCTACAAAAGCAAGACTTTAAACTTTTTAGATACATCATTAAAAA

*KRAS* p.Q61H. Fragment length: 999 base pairs

ATAAATAGTGCTGCTGCGAACATTGGTGTACATGTATCTGTTTGAGTCCCTGTTTTTAGTTATTTTGGTTATATACCTAGGAATGGAATTGCTGATCATATGGTAATTCTGTGTTTAACTTTTTGAGGAACTACCACTGTTTTCCACAATGGCATCACCATTTTACATTCCCACCAGCAATGCACAAAGATTTCAGTGTCTGTATCCTTGCTAACACTTATTTTCCATTTTTTGAGTTTTTTTGTTTTGTTTTTTTAATAATAGCCAATCCTAATGGGTATGTGGTAGCATCTCATGGTTTTGATTTTATTTTCCTGACTATTGATGATGTTGAGCATCTTTTCAGGTGCTTAGTGGCCATTTGTCCGTCATCTTTGGAGCAGGAACAATGTCTTTTCAAGTCCTTTGCCCATTTTTAAATTGAATTTTTTGTTGTTGAGTTGTATATAACACCTTTTTTGAAGTAAAAGGTGCACTGTAATAATCCAGACTGTGTTTCTCCCTTCTCAGGATTCCTACAGGAAGCAAGTAGTAATTGATGGAGAAACCTGTCTCTTGGATATTCTCGACACAGCAGGTCACGAGGAGTACAGTGCAATGAGGGACCAGTACATGAGGACTGGGGAGGGCTTTCTTTGTGTATTTGCCATAAATAATACTAAATCATTTGAAGATATTCACCATTATAGGTGGGTTTAAATTGAATATAATAAGCTGACATTAAGGAGTAATTATAGTTTTTATTTTTTGAGTCTTTGCTAATGCCATGCATATAATATTTAATAAAAATTTTTAAATAATGTTTATGAGGTAGGTAATATCCCTGTTTTATAAATGAAGTTCTTGGGGGATTAGAGCAGTGGAGTAACTTGCTCCAGACTGCATCGGTAGTGGTGGTGCTGGGATTGAAACCTAGGCCTGTTTGACTCCACAGCCTTCTGTACTCTTGACTATTCTACAAAAGCAAGACTTTAAACTTTTTAGATACATCATTAAAAA

*KRAS* p.K117N. Fragment length: 973 base pairs

GCACTTGTTATATTGAAAATGATTAACATGTAGAAGGGCTTTTAATGGAATAAGTGGTGTAGTAACTTCAGTGTTGCCTACCTAGAAATCAAAATCTTTCTAGTTGTCCACTTTGTTTTTTGAAAAAGTAATATGAAAATTATGTTAATGCTTTAATTCAGGTTTTTGTAAAATATTTTTTATCTTTACACATTTAACATACGTTTCTAAAATTATAGTCTGTTATATAGCACTTTGGGTCTAGAATTTTTCAGTAGTTTCTGTTTTACTATTATGATCTACCTGCATATTAACCTATTAGGTTATAGTTTTACTATACTTCTAGGTATTTGATCTTTTGAGAGAGATACAAGGTTTCTGTTTAAAAAGGTAAAGAAACAAAATAACTAGTAGAAGAAGGAAGGAAAATTTGGTGTAGTGGAAACTAGGAATTACATTGTTTTCTTTCAGCCAAATTTTATGACAAAAGTTGTGGACAGGTTTTGAAAGATATTTGTGTTACTAATGACTGTGCTATAACTTTTTTTTCTTTCCCAGAGAACAAATTAAAAGAGTTAAGGACTCTGAAGATGTACCTATGGTCCTAGTAGGAAATAATTGTGATTTGCCTTCTAGAACAGTAGACACAAAACAGGCTCAGGACTTAGCAAGAAGTTATGGAATTCCTTTTATTGAAACATCAGCAAAGACAAGACAGGTAAGTAACACTGAAATAAATACAGATCTGTTTTCTGCAAAATCATAACTGTTATGTCATTTAATATATCAGTTTTTCTCTCAATTATGCTATACTAGGAAATAAAACAATATTTAGTAAATGTTTTTGTCTCTTGAGAGGGCATTGCTTCTTAATCCAGTGTCCATGGTACTGCTTTTGGCTTTGGTTTCTTTCTACATTGAAAATTTCTCTTCAATTCTGAGCACATGTTAACATTTAGAATTCAAGAGGTGGGGATTTTTTTTTCCCATGGTTAC

*KRAS* p.A146V. Fragment length: 975 base pairs

GCACTTGTTATATTGAAAATGATTAACATGTAGAAGGGCTTTTAATGGAATAAGTGGTGTAGTAACTTCAGTGTTGCCTACCTAGAAATCAAAATCTTTCTAGTTGTCCACTTTGTTTTTTGAAAAAGTAATATGAAAATTATGTTAATGCTTTAATTCAGGTTTTTGTAAAATATTTTTTATCTTTACACATTTAACATACGTTTCTAAAATTATAGTCTGTTATATAGCACTTTGGGTCTAGAATTTTTCAGTAGTTTCTGTTTTACTATTATGATCTACCTGCATATTAACCTATTAGGTTATAGTTTTACTATACTTCTAGGTATTTGATCTTTTGAGAGAGATACAAGGTTTCTGTTTAAAAAGGTAAAGAAACAAAATAACTAGTAGAAGAAGGAAGGAAAATTTGGTGTAGTGGAAACTAGGAATTACATTGTTTTCTTTCAGCCAAATTTTATGACAAAAGTTGTGGACAGGTTTTGAAAGATATTTGTGTTACTAATGACTGTGCTATAACTTTTTTTTCTTTCCCAGAGAACAAATTAAAAGAGTTAAGGACTCTGAAGATGTACCTATGGTCCTAGTAGGAAATAAATGTGATTTGCCTTCTAGAACAGTAGACACAAAACAGGCTCAGGACTTAGCAAGAAGTTATGGAATTCCTTTTATTGAAACATCAGTAAAGACAAGACAGGTAAGTAACACTGAAATAAATACAGATCTGTTTTCTGCAAAATCATAACTGTTATGTCATTTAATATATCAGTTTTTCTCTCAATTATGCTATACTAGGAAATAAAACAATATTTAGTAAATGTTTTTGTCTCTTGAGAGGGCATTGCTTCTTAATCCAGTGTCCATGGTACTGCTTTTGGCTTTGGTTTCTTTCTACATTGAAAATTTCTCTTCAATTCTGAGCACATGTTAACATTTAGAATTCAAGAGGTGGGGATTTTTTTTTCCCATGGTTAC
